# A Novel Technique of Ultra-Mini-Percutaneous Nephrolithotomy: Introduction and an Initial Experience for Treatment of Upper Urinary Calculi Less Than 2 cm

**DOI:** 10.1155/2013/490793

**Published:** 2013-07-24

**Authors:** Janak Desai, Guohua Zeng, Zhijian Zhao, Wen Zhong, Wenzhong Chen, Wenqi Wu

**Affiliations:** ^1^Department of Urology, Samved Hospital, Ahmedabad 380009, India; ^2^Department of Urology, Minimally Invasive Surgery Center, The First Affiliated Hospital of Guangzhou Medical University, Guangdong Key Laboratory of Urology, Guangzhou 510230, China

## Abstract

*Objectives*. To describe our novel modified technique of ultra-mini-percutaneous nephrolithotomy (UMP) using of a novel 6 Fr mininephroscope through an 11–13 Fr metal sheath to perform holmium: YAG laser lithotripsy. *Methods*. The medical records of 36 patients with moderate-sized (<20 mm) kidney stones treated with UMP from April to July 2012 were retrospectively reviewed. Patients were assessed at the 1st day and 1st month postoperatively by KUB and US to assess stone-free status. *Results*. The mean stone size was 14.9 ± 4.1 mm (rang: 6–20). The average operative time was 59.8 ± 15.9 (30–90) min. The stone-free rate at postoperative 1st day and 1st month was 88.9% and 97.2%. The mean hospital stay was 3.0 ± 0.9 (2–5) days. Complications were noted in 6 (16.7%) cases according to the Clavien classification, including sepsis in 2 (5.6%) cases (grade II), urinary extravasations in 1 (2.8%) case (grade IIIa), and fever in 3 (8.3%) cases (grade II). No patients needed blood transfusion. *Conclusions*. UMP is technically feasible, safe, and efficacious for moderate-sized renal stones with an advantage of high stone-free rates and low complication rates. However, due to the limits of its current unexplored indications, UMP is therefore a supplement to, not a substitute for, the standard mini-PCNL technology.

## 1. Introduction

The treatment of urolithiasis has undergone a paradigm shift in the past decade. Management of urolithiasis necessitates a balance between stone clearance and morbidity related to the procedure. As a low-risk procedure with a high retreatment (18%~67%), ESWL often leads to persistent residual stones [1–3]. The developing retrograde intrarenal surgery (RIRS) can minimize the risks associated with bleeding and visceral injury, but sometimes the nonideal pelvicaliceal anatomy and poor durability of the flexible ureteroscopy may impact its success rate and applications [4–6]. 

Of the minimally invasive treatment strategies, the PCNL procedure is simply based on the creation of a proper percutaneous renal access, through the most appropriate part of the kidney, dilation of this tract, and fragmentation and removal of the stone fragments using the nephroscope through the access sheath. It has been reported that PCNL can be performed safely and effectively to achieve a higher stone-free rate and allow a short treatment period in most patients [1, 2, 7], despite its well-known hazardous and serious complications [8, 9]. Most of these complications are related to tract formation and size. Efforts to decrease the complications of PCNL have focused on access size. After enough evidence in the literature suggested that decreasing the tract size for PCNL could decrease bleeding and morbidity, Desai et al. developed an all-seeing needle and used it in a 4.85 Fr tract size without a working sheath to perform PCNL, which was called “microperc” [10, 11]. 

Here, we adopted a new 6 Fr Mininephroscope with some special features that allow the performances of PCNL in an 11–13 Fr metal sheath. We termed the procedure as ultra-mini-percutaneous nephrolithotomy (UMP) because of its smaller tract compared to mini-PCNL. We reviewed our initial experience with the first 36 consecutive patients with moderate-sized (<20 mm) kidney stones to undergo the UMP.

## 2. Patients and Methods

### 2.1. Patients and Measurements

We obtained approval for this clinic study from the Ethics Committee of The First Affiliated Hospital of Guangzhou Medical University. Written informed consent that this was a trial of new surgical technique was obtained from all participants before operation. From April 2012 to July 2012, UMP was performed on 36 patients by one of the two experienced surgeons (G. Zeng and J. Desai). Inclusion criteria included all patients with kidney stone size less than 20 mm who preferred to undergo UMP. The primary aim of the study was to introduce the UMP technique (including equipment required, renal access method, number of punctures required, tract dilation method, stone fragmentation, and extraction method) and to report the outcome of UMP in this group patients (including intraoperative and postoperative complications, operative time, hemoglobin decrease, hospital stay, stone-free rate, and the need for auxiliary procedures). 

A detailed medical history, physical examination, urinalysis, urine culture, complete blood count, serum biochemistry, coagulation test, kidney urinary bladder X-ray (KUB), renal ultrasonography (US), and/or intravenous urography (IVU) were performed on all patients. The computed tomography (CT) was used only in special situations because of the concerns about the radiation exposure. Patients who had positive urine cultures were treated with appropriate antibiotics immediately. Stone size was calculated by measuring the longest axis on preoperative imaging. The composition of the stones of all patients was analyzed by infrared spectroscopy. Stone-free status was assessed at the 1st day and 1st month postoperatively and defined as no residual stone on KUB and ultrasound [11]. Perioperative complications of all patients were recorded according to the modified Clavien classification system [9]. Hospital stay was rounded to the nearest whole day and calculated from the date of hospitalization to the date of discharge home. Data were reported as the number and percent or mean ± SD as appropriate see Table 2.

### 2.2. Armamentarium

**Figure 1:**
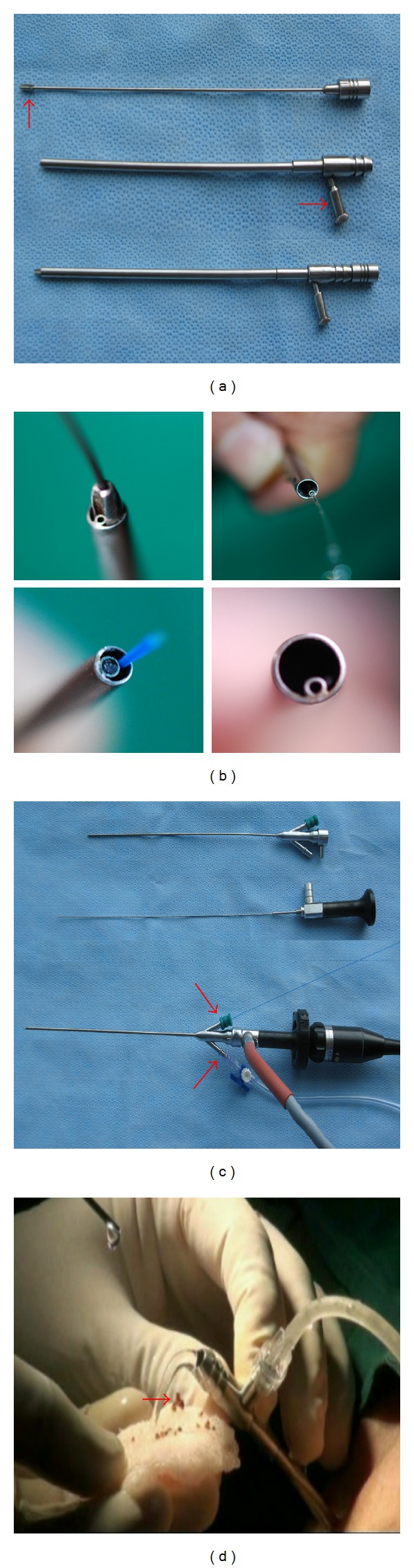
Special basic instruments set of the UMP. (a) Working metal sheath and its obturator. (b) Spatial location of the fine tube, obturator, guide wire and laser fiber, and water jet out from inner fine tube. (c) Mininephroscope assembled with a 3.5 Fr telescope and a 6 Fr demountable telescope (inner) sheath. The 3.5 Fr telescope passes through the central channel. Irrigation connection and laser fiber are each attached to one side port. Optics is connected to a zoom ocular and light adapter. (d) Water jet flush out some larger stone debris (<3 mm) and blood clots through the working sheath after rapid removal of the endoscope.

A Mininephroscope and working sheath were designed at our institute and fabricated locally. One of the special basic instruments set of the UMP was an 11 Fr or 13 Fr metal sheath with an integrated side port (Figure 1(a)). It has a housed 1 mm diameter of inner fine tube (LUT-GmbH, Germany) (Figure 1(b)). There is a channel from the inner fine tube to the side port, and the side port could attach a syringe to produce a water jet (Figure 1(b)). The metal sheath could be matched with an obturator inside itself. The obturator was beveled with a side groove in the distal end, so the guide wire could pass through the obturator (Figure 1(b)). Another special basic instrument was a 3.5 Fr telescope with a 6 Fr demountable telescope sheath (LUT-GmbH, Germany) (Figure 1(c)), and the telescope sheath has two side ports. The assembled 6 Fr telescope is used for visualization. One side port of the telescope sheath is connected to an irrigation pump for irrigation and the other side port is used to pass the laser fiber. Optics is connected via a zoom ocular and light adapter to a standard endoscopic camera system (Figure 1(c)).

### 2.3. UMP Technique

All procedures were performed under continual epidural anesthesia. After retrograde ureteric catheterization with a 5-6 Fr open-ended ureteric catheter (Boston Scientific Corporation, Miami Technology Center, USA), all other procedures were performed in the prone position with a pack under the abdomen to minimize lumbar lordosis. Fluoroscopy-guided percutaneous punctures were made by the urologist, followed by passage of an 18-gauge coaxial needle (Cook Incorporated, Bloomington, IN, USA) into the designed posterior or anterior calyx after retrograde injection of contrast medium. Fluoroscopy was used to guide the puncture; the line of the puncture holder was directed toward the calyx. If the puncture holder was unavailable, the pathway of the puncture needle was identified under the real-time ultrasonic monitor. After the fluid efflux was seen or the urine was aspirated, the diluted (30%–50%) contrast medium was injected into the collecting system to confirm the puncture. Once the needle was properly placed into the calyx, a 0.028 inch flexible tip Zebra guide wire (Boston Scientific Corporation, Miami Technology Center, USA) was then inserted into renal collecting system or down the ureter through the needle sheath under fluoroscopic guidance. Then, the needle was retracted. Then, the 11 Fr or 13 Fr metal working sheath assembled with an obturator was gradually advanced over the guide wire to an appropriate location in the desire calyx. Next, the obturator is removed, and the sheath is still placed as a working sheath. Subsequently, the assembled 6 Fr telescope is inserted to the working mental sheath to inspect the collecting system. 

The calculus is then fragmented by a holmium:YAG laser using a 200 or 365 um holmium laser fiber with the settings of 10–20 W under direct visualization. The maintenance of the vision was provided by an endoscopic pulsed perfusion pump controlled by the surgeon. The perfusion pump used in UMP could be the same in mini-PCNL. In our center, the irrigation pump (MMC Yiyong, Guangzhou, China) was designed by us, and it can generate pressure up to 300 mmHg for about 3 seconds, then stop for 2 seconds, and then repeat the cycle. In the UMP procedure, 100~150 mmHg pressure was adequate. During the procedures, we can also manually use a syringe to inject sterile saline solution via the side port of the metal sheath to produce a water jet to improve the visualization, if needed. During stone fragmentation, rapid removal of the endoscope out of the working sheath synchronized with the water jet period would create a relative vacuum within the working sheath and, together with the recoil of water jets, would flush out the small stone fragment (≤3 mm) and blood clots (Figure 1(d)). Also, the tiny stone dust and fragments could be simply flushed or jettisoned down the ureter to discharge out of the body. 

At the end of the procedure, the collecting system accessible to the UMP telescope combined fluoroscopy was examined for potential fragments. No double-J stent was routine placed at the end of the procedure except for patients with amounts of remaining small stone debris. No silastic nephrostomy tube (Boston Scientific, Natick, MA, USA) was routine placed except for needing a second-look UMP. Second-look UMP was recommended for some cases with the following situations: intraoperative significant hemorrhage which leads to an obscure operative field. If the patients placed the double-J ureteral stent, it was removed 1 week after the procedure. Hemoglobin was tested during postoperative 24 hours. Routine KUB and retrograde nephrostogram were done in all patients to assess for residual stone fragments, urinary leakage, and infrarenal obstruction after postoperative day 1. Patients were discharged home 1 day after the procedure, provided that no significant residual stone was seen on the KUB. KUB or ultrasound is also done in a 1-month followup to assess the stone-free rate in all patients. 

## 3. Results

Table 1 lists patients and stone characteristics. Mean age of 36 consecutive patients was 48.2 years (range 2 to 79). The mean BMI was 24.63 (18.1–33.2) kg/m^2^. The mean preoperative stone size was 14.9 mm (range 6 to 20). The indication of UMP in patients of this study included transplant kidney in 1, solitary kidney in 1, narrow infundibulum in 1, diverticular renal stones in 3, preschool children in 2, refractory ESWL in 6, failed RIRS in 5 (3 lower pole calculi with acute infundibular-pelvic angle and 2 diverticulum stones where calyceal neck could not be opened during RIRS), and the rest of the patients with stone size less than 20 mm preferred the novel technique. 

All 36 patients were treated with a single tract. In 15 patients, access was obtained via a supracostal puncture, while in 21, each access was achieved via an infracostal puncture, respectively. Moreover, although most of the punctures were through the posterior calyx in 30 patients, the anterior calyx puncture was tentative in application in order to directly reach the target stone calyx in 6 patients. No patients had to be converted to a conventional method or abandoned. Mean operative time was 59.8 minutes (range 30 to 90). The mean hemoglobin decrease was 5.4 g/L (range 0 to 21), and no one needed the blood transfusion.

Of the patients, 32 (88.9%) had no residual calculi on postoperative KUB, 2 (9.7%) had residual fragments with 3-4 mm, and 2 (4.9%) had residual stone fragments greater than 4 mm, and one of the 2 needed a second-look procedure after 2 days of the initial UMP session. At the 1 month followup, stone-free status of 97.2% (35/36) was achieved; whereas 1 patient remained a 5 mm asymptomatic calculus in lower pole calyx accepted conservative observation. 

According to the modified Clavien classification system, complications were noted in 6 (16.7%) cases. Urosepsis (grade II) with a slight longer operative time (87 min) occurred in 2 (5.6%) cases and fever (grade II) occurred in 3 (8.3%) cases, which were all cured by intravenous antibiotics and/or Double-J stent indwelling. Urinary extravasations (grade IIIa) occurred in 1 (2.8%) case because excessive fragments moved down to the ureter resulting in obstruction, which was cured by inserting a Double-J stent. No patients underwent significant bleeding even in 6 patients with an anterior calyx approach. No patients needed blood transfusion. No pneumothorax occurred in the 15 patients who underwent a supracostal approach.

Mean postoperative hospital stay was 3 days (range 2 to 5). In the 29 patients with no residual stones and complications, mean of each hospital stay was 2.3 day. Postoperative fevers occurred in 3 patients, and the hospital stay of each patient was 3 days. In the 3 patients with urosepsis (2 cases) and urinary extravasations (1 case), respectively, requiring Double-J stent indwelled, each hospital stay was 4 days, respectively. In 1 patient requiring a second-look procedure, the hospital stay was 5 days, and this patient had a solitary kidney with a 20 mm stone, and a minor bleeding and slight longer operative time (90 minutes) were contributed to a termination of initial surgery.

Chemical analysis of stone composition was available in all 36 patients. Chemical analysis revealed pure calcium oxalate in 6 patients, pure calcium phosphate in 3, a mixture of calcium oxalate and calcium phosphate in 9, pure uric acid in 4, and struvite in 1. The remaining 13 patients had various other mixtures of stone types.

## 4. Discussion

Urolithiasis is a worldwide problem in the general population, due to its high prevalence and frequency of recurrence [12]. At present, various minimally invasive treatment strategies have been recommended to treat the urinary tract stones including ESWL, standard PCNL, mini-PCNL, RIRS, and a new emerging technique termed “micro-PCNL.” For the principle of stones treatment, there is a need for a surgical method that will allow both high stone-free rate and short treatment times, without the increased risk of hemorrhage. 

The location of the stone and pelvicalyceal system anatomy are the factors affecting the selection of the appropriate treatment regimen. Although the noninvasive nature, minimal anesthesia requirement, and high acceptance rate by the patients and physicians are main advantages of ESWL, low stone-free and higher retreatment rates are considered as the drawbacks. The reported stone-free rates of ESWL at three months for stones are 86% to 89% (renal pelvis), 71% to 83% (upper calyx), and 73% to 84% (middle calyx) and for lower pole stones based on stone size are 63% to 74% (1–10 mm), 23% to 56% (11–20 mm), and 14% to 33% (21–30 mm) [2]. RIRS has been popularized with the advances in endoscope and lithotripter technology. However, the collecting system anatomy restricts its success rate, and the high sustainable cost and poor durability restrict its wide application in most basic-level hospitals [4–6]. The lower calyceal fragment re-accumulation may be a cause of stone recurrence [3].

However, conventional percutaneous nephrolithotomy (PCNL) can cause a somewhat higher associated morbidity despite the fact that it achieves a higher stone-free rate and allows a short treatment period [1, 2, 7–9]. Most of these complications are related to tract formation and size. Efforts to decrease the complications of PCNL have focused on access size. How small of the tract size was the endpoint in PCNL technique? Theoretically, a smaller tract gives rise to fewer complications. After enough evidence in the literature suggested that decreasing the tract size could decrease bleeding and morbidity without affecting success rate, Desai et al. developed a 4.85 Fr all-seeing needle and used it in a 4.85 Fr tract size without a working sheath to perform PCNL, which was called “microperc” [10, 11]. 

During the development of the PCNL technique, the different terminology of it came out mainly according to the tract size such as standard, mini-, and micro-PCNL. Here, we modify an innovative PCNL, which is termed as ultra-mini-PCNL (UMP). The quintessential element of the novel UMP is using a novel 6 Fr Mininephroscope through an 11–13 Fr metal sheath to perform holmium:YAG laser lithotripsy. Dilation is achieved in one step with much less fluoroscopy time, and the cross-section of the puncture channel is only approximately 30% of that required with the conventional mini-PCNL (reference to 18 Fr size). This miniaturization is the main reason why no blood transfusion and why no nephrostomy tube routinely placed in this group of patients. These results match those of Jackman et al. who also observed no significant bleeding and suggested that the nephrostomy tube was unnecessary [13]. 

Both micro-PCNL and UMP do not allow stone fragments to be retrieved by forceps during PCNL. This may increase the operative time to disintegrate the stones. However, during stone fragmentation by UMP, the stones may not to be smashed to dust, while it disintegrates to a size less than 3 mm is ok. Then, like the mini-PCNL, small fragmented stones (≤3 mm) will pass around the endoscope and wash out through the sheath, whereas there is no capability of stone fragment retrieval via 4.8 Fr sheath during microperc. However, it is hard to make a decision that the immediately stone-free rate can be increased and the operative time can be shorten compared to micro-PCNL due to lacking data in this study. Secondly, intraoperative bleeding (leading to impairment of vision) and renal parenchyma injury may occur due to needle insertion again and again in micro-PCNL procedures, while a working sheath may diminish parenchymal bleeding in UMP procedures. Furthermore, if the ultra-mini-access is insufficient for the procedure, conversion to standard 14–20 Fr tract of mini-PCNL by extending the dilation is simple, as the tract access has already been established. This should be possible without a significant increase in the operative time required.

Compared to mini-PCNL or standard PCNL, anterior calyx puncture can be more attempted without much fear of vessel injury when UMP procedure was performed. No major bleeding occurred, especially puncture of one patient through a narrow infundibulum. Easy and safe supracostal puncture with few traumas to diaphragm was also observed in UMP just like in other versions of PCNL. No pneumothorax occurred in the 15 patients who underwent a supracostal approach. Blood vessel damage was minimal by UMP, as we know that a smaller tract causes less bleeding. 

UMP also would be an alternative to SWL or flexible ureteroscopy to suffer the less impact of pelvicaliceal anatomy. UMP provides the ability to gain direct access to the desired calyx and accomplish a higher immediately stone-free rate (88.9% in the present study). As results, the UMP would shoulder the responsibility for RIRS and ESWL failure cases.

We successfully performed UMP tentatively in 2 preschool children to prove its benefit in pediatric. The small size of the kidney and the more compact collecting system of infants necessitate the use of the smallest and least traumatic instruments possible to reduce the likelihood of major complications, such as bleeding and renal trauma [14, 15]. Bilen et al. demonstrated a higher transfusion need in children treated with tracts sized 20 to 26 Fr, whereas no transfusion was needed in the cohort treated with 14 Fr access tract [15]. UMP has a smaller access tracts than 14 Fr, which may be safer for pediatric by reducing the risk of bleeding and morbidity associated with tract size.

There are anticipated concerns regarding the limitations of this technique. First, in our study, 3 patients were taken nearly a 90-minute operative time. The operative time taken to fragment larger stones to a size enough to be extracted from the 11–13 Fr sheath was longer than the mini-PCNL. Secondly, in the past, the smaller working tract warned about the elevation of intrarenal pressure, which would induce the risks of bacteremia and sepsis. We once inspected that during mini-PCNL within the 14, 16, and 18 Fr percutaneous tracts, the average renal pelvic pressures were 24.85, 16.23, and 11.68 mmHg, respectively, all lower than the level required for a backflow (30 mmHg) [16]. The fluid in the pelvicalyceal system can be discharged easily. Already the inner diameter of the working sheath is much wider than the outer diameter of the Mininephroscope, and this situation may contribute to reduce the intrarenal pressure due to leakage from the edge of the working sheath. This remains to be proven and can gauge the pelvis pressure that the authors will publish in future.

In conclusions, we have performed 36 ultra-mini-PCNL procedures, and the initial results are promising. UMP is technically feasible, safe, and efficacious and an alternative for small volume renal calculous disease with an advantage of high immediate and final stone-free and lower complication rates. The indications for UMP are moderate-sized stones as an alternative to ESWL or RIRS, low pole stones which were not amenable to RIRS, diverticular renal stones, and stones refractory ESWL. Compared with ESWL and RIRS, the UMP increases the stone-free rate without increasing the complications. However, the limits of the UMP were that the operative time taken to fragment larger stones to a size enough to be extracted from the 11–13 Fr sheath was longer than that of the mini-PCNL. UMP is therefore a procedure that supplements to standard mini-PCNL and that cannot replace it. Further clinical and comparison of studies with available modalities are required to define the significance of UMP in relation to mini-PCNL in the treatment of kidney urolithiasis.

## Figures and Tables

**Table 1:** Patient demographics and stone characteristics.

Mean ± SD age (yr)	48.2 ± 15.7 (2–79)
No. male/female	23 (63.9%)/13 (36.1%)
Mean ± SD stone area (mm^2^)	160.6 ± 82.7 (30–380)
Mean ± SD stone size (mm)	14.9 ± 4.1 mm (6–20)
Left/right side stone	15 (41.7%)/21 (58.3%)
No. positive preoperative urine culture	None
No. medical comorbidity	
Hypertension	2 (5.6%)
Diabetes	2 (5.6%)
Renal failure	1 (2.8%)
Spinal disease	1 (2.8%)
Poly renal cyst	1 (2.8%)
Stone locations	
Pelvic calculi	5 (14%)
Upper calyceal stone	7 (19.4%)
Middle calyceal stone	8 (22.2%)
Lower calyceal stone	10 (27.8%)
Upper ureteral stone	3 (8.3%)
Multiple calyceal stones	3 (8.3%)
Patients with special situations	
Transplanted kidney stone	1 (2.8%)
Diverticular renal stone	2 (5.6%)
Stone with narrow infundibulum	1 (2.8%)
Residual stones after ESWL	6 (16.7%)
Failed RIRS	5 (13.9%)
Residual stones after PCNL	4 (11.2%)
Preschool children	2 (5.6%)
Solitary kidney	1 (2.8%)

**Table 2:** Intraoperative and postoperative parameters.

Immediate stone-free rate (after 1 day)	88.9% (32/36)
Total stone-free rate (after 1 month)	97.2% (35/36)
Hospital stay (day)	3.0 ± 0.9 (2–5)
No. Double-J stent (%)	4 (11.1%)
Operative time (min)	59.8 ± 15.9 (30–90)
Hemoglobin drop (g/L)	5.4 ± 7.8 (0–21)
Second-look UMP	1 (2.8%)
No. of tract size 13Fr/11Fr	31 (86.1%)/5 (13.9%)
No. of puncture locations	
Upper/middle/lower pole calyces	7 (19.4%)/15 (41.7%)/ 14 (38.9%)
Supracostal/infracostal	15 (41.7%)/21 (58.3%)
Posterior/anterior calyx	30 (83.3%)/6 (16.7%)
Significant complication	6 (16.7%)
Urosepsis	2 (5.6%)
Urinary extravasations	1 (2.8%)
Fever (>38.5°C)	3 (8.3%)
Stone analysis	
Calcium oxalate and/or phosphate	18 (50%)
Struvite	1 (2.8%)
Uric acid	4 (11.1%)
Mix stones	13 (36.1%)
